# Supplementary material for: Microbiome–Metabolome Reveals the Contribution of the Gut–Testis Axis to Sperm Motility in Sheep (Ovis aries)
Source: Animals (Basel). 2023 Mar 9;13(6):996. doi: 10.3390/ani13060996 (PMC10044597; doi:10.3390/ani13060996)
Supplement: Supplementary file 1 [file animals-13-00996-s001.zip › animals-2238989-supplementary.pdf]

Supplementary Material

Table S1 | The motility parameters of sheep sperm were tested

	HM(mean±SEM)	LM(mean±SEM)	*p* value
Concentration(million/ml)	379.9±48.5[a]	267.4±52.5[b]	0.0190
Sperm motility(%)	94.4±1.93[a]	43.8±3.74[b]	<0.0001
VCL(μm/s)	135.3±4.13[a]	109.3±1.76[b]	0.0012
VSL(μm/s)	35.1±1.86[a]	21.4±4.15[b]	<0.0001
VAP(μm/s)	66.7±2.64[a]	40.6±9.46[b]	<0.0001
WOB(%)	54.03±2.76[a]	63.8±3.07[b]	0.0287
BCF(Hz)	6.7±0.09[a]	6.0±0.22[b]	0.0117

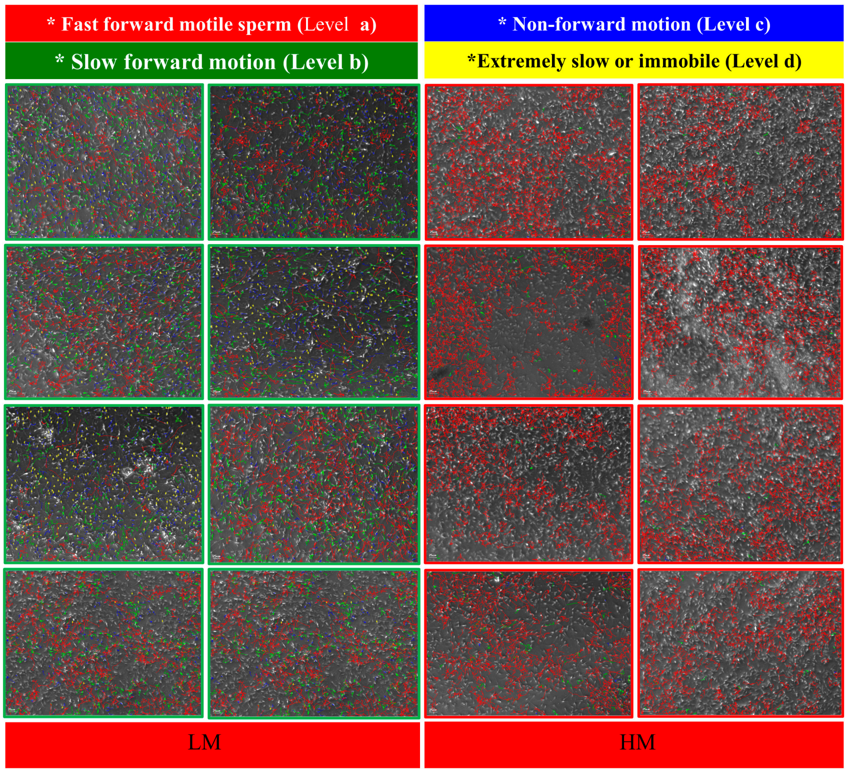

Figure S1 The state of sperm quality was observed in the LM and HM groups under the computer-aided Sperm Analysis system (CASAS)

Table S2 Significantly differentiated genera between the LM and HM groups (with $p < 0.05$ by Wilcoxon rank-sum test. The microbes present at more than 0.1% relative abundance were shown).

Taxa (Phylum)	Genus	HM (Mean±SE)	LM (Mean±SE)	*P*-value
Firmicutes	Quinella	22.45±2.48E+00	12.35±1.37E+00	0.03
	Ruminococcus	4.19±4.64E-01	1.76±1.95E-01	0.03
	Christensenellaceae_R_7_group	3.70±4.10E-01	2.10±2.32E-01	0.03
	Anaeroplasma	3.52±3.90E+01	0.43±4.78E+00	0.03
	UCG_004	0.57±6.32E+00	1.34±1.49E+01	0.03
	Saccharofermentans	0.30±3.33E+00	0.71±7.83E+00	0.03
	Erysipelotrichaceae_UCG_009	0.55±3.54E+00	0.56±6.25E+00	0.03
	unclassified_Lachnospiraceae	1.36±1.50E+01	0.50±5.56E+00	0.03
	Lachnospiraceae_AC2044_group	1.30±1.43E+01	0.42±4.60E+00	0.00
	Lachnospiraceae_XPB1014_group	0.97±1.07E+01	0.12±1.36E+00	0.03
Bacteroidota	Rikenellaceae_RC9_gut_group	8.58±9.50E-01	10.58±1.17E+00	0.03
	Prevotellaceae_UCG_001	0.76±8.41E+00	0.39±4.35E+00	0.03
Synergistota	Fretibacterium	2.42±2.68E-01	5.03±5.57E-01	0.03
Actinobacteriota	unclassified_Bifidobacteriaceae	1.62±1.79E+01	0.50±5.55E+00	0.03
Proteobacteria	Succinivibrionaceae_UCG_002	1.29±1.43E+01	0.24±2.70E+00	0.03

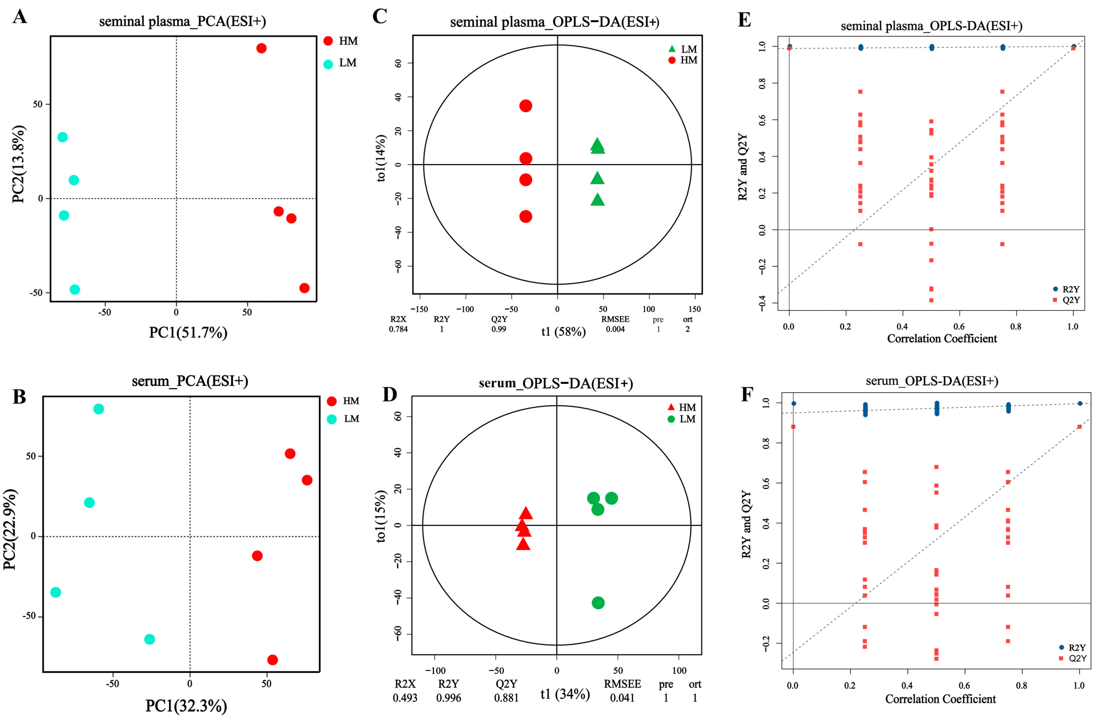

Figure S2 Serum and seminal plasma metabolomic profile in different groups. (**A,B**)PCA (positive ion mode) score plots of seminal plasma and serum metabolic profiling of LM and HM groups.(**C,D**) OPLS-DA (positive ion mode) score plots of seminal plasma and serum metabolic profiling of LM and HM groups.(**E,F**) The permutations plots of the OPLS-DA models. LM,low motility;HM,high motility.

Table S3 Differential metabolites found in seminal plasma samples

Metabolite	HMDB	KEGG	Fold Change (FC=2)	Pvalue	VIP	ESI	Content Level
Eicosatetraenoic acid	-	-	7.85	1.29E-03	1.28	–	CLM<CHM
Catechol	-	C00090	5.46	2.85E-04	1.24	–	CLM<CHM
L-Histidine	HMDB0000177	C00135	4.27	9.79E-08	1.30	–	CLM<CHM
D-Lactic acid	HMDB0001311	C00256	3.68	3.52E-05	1.30	–	CLM<CHM
D-Leucic acid	HMDB0000624	C03264	2.95	5.61E-04	1.26	–	CLM<CHM
L-Glutamate	HMDB0000148	C00025	2.88	2.02E-05	1.30	–	CLM<CHM
Raffinose	HMDB0003213	C00492	2.65	1.14E-05	1.30	–	CLM<CHM
Pantothenic Acid	HMDB0000210	C00864	2.63	1.40E-05	1.29	–	CLM<CHM
Tryptamine	HMDB0000303	C00398	2.36	6.69E-04	1.25	–	CLM<CHM
L-Tryptophan	HMDB0000929	C00078	2.29	2.53E-04	1.27	–	CLM<CHM
D-Mannitol	HMDB0000765	C00392	2.07	3.07E-08	1.30	–	CLM<CHM
Saccharin	HMDB0029723	C12283	0.41	5.10E-03	1.14	–	CLM>CHM
Indole-3-carbinol	HMDB0005785	-	0.38	2.18E-02	1.02	–	CLM>CHM
(+)-Nicotine	HMDB0001934	C00745	0.37	6.89E-04	1.22	–	CLM>CHM
L-Valine	HMDB0000883	C00183	0.36	1.48E-04	1.30	–	CLM>CHM
Riboflavin	HMDB0000244	C00255	0.35	6.44E-04	1.28	–	CLM>CHM
2-Arachidonoylglycerol	-	-	0.31	1.10E-03	1.27	–	CLM>CHM
Naringenin	HMDB0002670	C00509	0.11	1.85E-05	1.30	–	CLM>CHM
Creatinine	HMDB0000562	C00791	0.01	2.46E-02	1.13	–	CLM>CHM
Quinine	HMDB0014611	C06526	0.01	3.13E-04	1.29	–	CLM>CHM
Zearalanone	-	-	0.00	3.35E-02	1.09	–	CLM>CHM
Neomycin	HMDB0015129	C01737	0.22	8.34E-05	1.27	–	CLM>CHM
Oxytocin	HMDB0002865	C00746	6.03	8.08E-06	1.31	+	CLM<CHM
Zeranol	HMDB0032702	C14752	5.16	4.72E-03	1.21	+	CLM<CHM
Daidzein	HMDB0003312	C10208	4.47	1.93E-05	1.31	+	CLM<CHM
Estreptoquinasa	-	-	2.05	7.72E-04	1.29	+	CLM<CHM
Fluvastatin	HMDB0015227	C07014	2.19	1.43E-02	1.17	+	CLM<CHM
Lamivudine	HMDB0014847	C07065	3.41	2.91E-02	1.03	+	CLM<CHM
Capsaicin	HMDB0002227	C06866	0.45	2.39E-04	1.29	+	CLM>CHM
oxymetholone	-	-	0.42	2.28E-05	1.29	+	CLM>CHM
3',5'-Cyclic AMP	-	-	0.37	8.08E-04	1.25	+	CLM>CHM
ANETHOLE	HMDB0030837		0.23	2.04E-02	1.10	+	CLM>CHM
Everolimus	HMDB0015529		0.21	1.90E-03	1.26	+	CLM>CHM
Solasodine	HMDB0035282	C10822	0.19	8.87E-05	1.30	+	CLM>CHM
Hexanoyllysine	-	-	0.13	1.81E-04	1.31	+	CLM>CHM
Androstenedione	HMDB0000053	C00280	0.06	1.18E-05	1.29	+	CLM>CHM
Caffeine	HMDB0001847	C07481	0.03	1.50E-04	1.31	+	CLM>CHM
Gemcitabine	HMDB0014584	C07650	0.01	1.05E-03	1.29	+	CLM>CHM
6-Hydroxymelatonin	HMDB0004081	C05643	0.31	9.60E-04	1.26	+	CLM>CHM

Table S4 Differential metabolites found in serum samples

Metabolite	HMDB	KEGG	Fold Change (FC=1.5)	Pvalue	VIP	ESI	Content Level
(S,E)-Zearalenone	HMDB0031752	C09981	77.28	3.77E-02	1.28	–	$C_{LM}<C_{HM}$
Gemfibrozil	HMDB0015371	C07020	5.75	1.67E-04	1.50	–	$C_{LM}<C_{HM}$
Eicosopentanoic acid	-	-	4.61	3.08E-02	1.28	–	$C_{LM}<C_{HM}$
Aciclovir	HMDB0014925	C06810	2.51	5.80E-04	1.54	–	$C_{LM}<C_{HM}$
cAMP	HMDB0000058	C00575	1.92	2.79E-02	1.19	–	$C_{LM}<C_{HM}$
Cannabidiol	-	C07578	1.87	3.44E-03	1.42	–	$C_{LM}<C_{HM}$
Allopurinol	HMDB0014581	-	1.74	7.98E-05	1.52	–	$C_{LM}<C_{HM}$
Riboflavin	HMDB0000244	C00255	1.71	3.98E-05	1.54	–	$C_{LM}<C_{HM}$
Topotecan	HMDB0015164	C11158	1.58	1.38E-03	1.45	–	$C_{LM}<C_{HM}$
Dopamine	HMDB0000073	C03758	1.56	4.32E-04	1.50	–	$C_{LM}<C_{HM}$
Saccharin	HMDB0029723	C12283	0.62	2.50E-02	1.32	–	$C_{LM}>C_{HM}$
Arachidic acid	HMDB0002212	C06425	0.59	3.05E-03	1.45	–	$C_{LM}>C_{HM}$
Sphingosine 1-phosphate	HMDB0000277	C06124	0.53	6.05E-04	1.52	–	$C_{LM}>C_{HM}$
heptanoate	HMDB0000666	C17714	0.49	1.53E-03	1.54	–	$C_{LM}>C_{HM}$
Calcitetrol	HMDB0006228	C18231	0.47	1.22E-05	1.55	–	$C_{LM}>C_{HM}$
Lutein	HMDB0003233	C08601	0.44	2.55E-02	1.23	–	$C_{LM}>C_{HM}$
Secoisolariciresinol	HMDB0013692	C18167	0.43	4.03E-03	1.51	–	$C_{LM}>C_{HM}$
Arachidoyl Ethanolamide	-	-	0.34	2.17E-02	1.31	–	$C_{LM}>C_{HM}$
Stanozolol	HMDB0003116	C07311	0.31	1.37E-02	1.35	–	$C_{LM}>C_{HM}$
15-Deoxyprostaglandin J2	HMDB0005079	C14717	0.24	2.45E-02	1.32	–	$C_{LM}>C_{HM}$
Simvastatin	-	-	0.24	2.49E-02	1.33	–	$C_{LM}>C_{HM}$
Crocetin	-	-	0.22	2.16E-02	1.30	–	$C_{LM}>C_{HM}$
Dihydroxyeicosatrienoic acid	-	-	0.03	1.72E-02	1.40	–	$C_{LM}>C_{HM}$
Mibefradil	-	-	0.00	5.22E-04	1.55	–	$C_{LM}>C_{HM}$
Exemestane	HMDB0015125	C08162	2.19	5.85E-03	1.49	+	$C_{LM}<C_{HM}$
(±)-Propionylcarnitine	-	-	2.19	4.40E-04	1.67	+	$C_{LM}<C_{HM}$
(-)-Riboflavin	HMDB0000244	C00255	2.09	4.70E-03	1.59	+	$C_{LM}<C_{HM}$
Ranitidine	HMDB0001930	D00673	2.01	5.47E-03	1.49	+	$C_{LM}<C_{HM}$
Estradiol-17beta	HMDB0000151	C00951	1.76	3.27E-02	1.36	+	$C_{LM}<C_{HM}$
1,3-Butadiene	HMDB0041792	C16450	1.54	1.91E-02	1.37	+	$C_{LM}<C_{HM}$
Celastrol	HMDB0002385		0.45	3.63E-02	1.40	+	$C_{LM}>C_{HM}$
Glycerol tripropanoate	HMDB0032857		0.37	2.42E-02	1.42	+	$C_{LM}>C_{HM}$
Cytochalasin B	-	-	0.32	8.86E-06	1.70	+	$C_{LM}>C_{HM}$
Arbutin	HMDB0029943	C06186	0.17	3.57E-02	1.31	+	$C_{LM}>C_{HM}$
Amphotericin B	HMDB0014819	C06573	0.00	3.55E-02	1.41	+	$C_{LM}>C_{HM}$

Table S5 Results of seminal plasma sample pathway analysis

Pathway	Total	Hits	p	-log10(P)	FDR	Impact
Aminoacyl-tRNA biosynthesis	48	4	9.12E-05	4.0399	0.0076621	0
Histidine metabolism	16	2	0.0034495	2.4622	0.28631	0.22131
Pantothenate and CoA biosynthesis	19	2	0.0048712	2.3124	0.39944	0.00714
Tryptophan metabolism	41	2	0.021854	1.6605	1	0.18296
Riboflavin metabolism	4	1	0.023046	1.6374	1	0.5
Nitrogen metabolism	6	1	0.034392	1.4635	1	0
D-Glutamine and D-glutamate metabolism	6	1	0.034392	1.4635	1	0.5
Valine, leucine and isoleucine biosynthesis	8	1	0.04562	1.3408	1	0

Table S6 Results of serum sample pathway analysis

Pathway	Total	Hits	p	-log10(P)	FDR	Impact
Riboflavin metabolism	4	2	0.0018472	2.7335	0.15516	0.5
Glycerophospholipid metabolism	36	3	0.025307	1.5968	0.79496	0.26172
Pyrimidine metabolism	39	3	0.031243	1.5052	0.79496	0.1047
Steroid biosynthesis	42	3	0.037855	1.4219	0.79496	0.16506

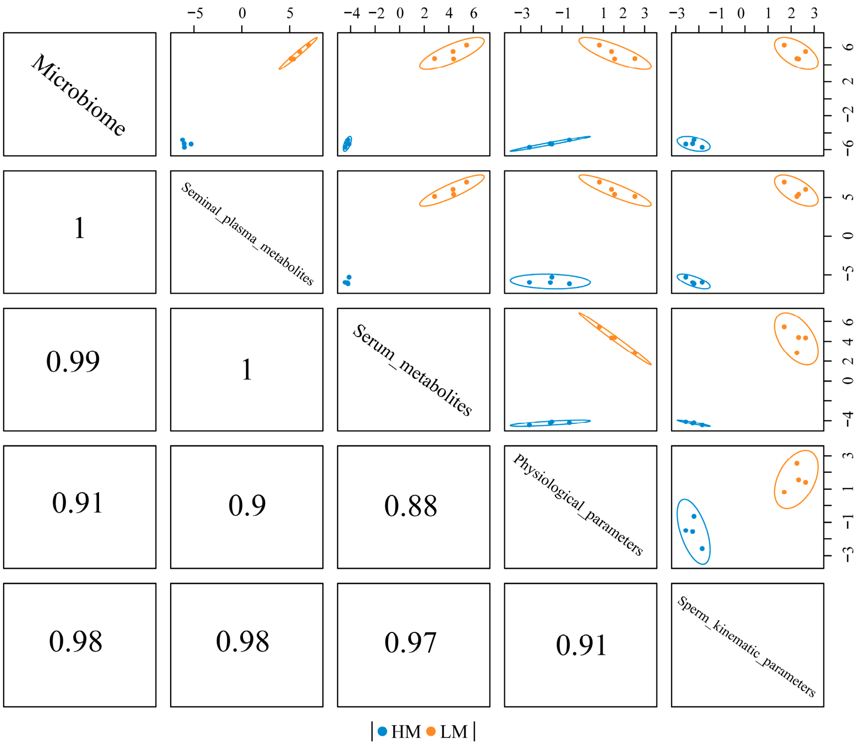

Figure S3 Multi-omics integrated analysis of DIABLO
